# Acute exposure to organic and inorganic sources of copper: Differential response in intestinal cell lines

**DOI:** 10.1002/fsn3.857

**Published:** 2018-10-20

**Authors:** Joanne Keenan, Finbarr O'Sullivan, Michael Henry, Laura Breen, Padraig Doolan, Indre Sinkunaite, Paula Meleady, Martin Clynes, Karina Horgan, Richard Murphy

**Affiliations:** ^1^ National Institute for Cellular Biotechnology Dublin City University Dublin Ireland; ^2^ Alltech Dunboyne Ireland

**Keywords:** aggresome formation, misfolded proteins, nutrient copper sources, proteomic changes

## Abstract

**Scope:**

Copper supplementation in nutrition has evolved from using inorganic mineral salts to organically chelated minerals but with limited knowledge of the impact at the cellular level.

**Methods:**

Here, the impact of inorganic and organic nutrient forms (glycinate, organic acid, and proteinate) of copper on the cellular level is investigated on intestinal cell lines, HT29 and Caco‐2, after a 2‐hr acute exposure to copper compounds and following a 10‐hr recovery.

**Results:**

Following the 10‐hr recovery, increases were observed in proteins involved in metal binding (metallothioneins) and antioxidant response (sulfiredoxin 1 and heme oxygenase 1), and global proteomic analysis suggested recruitment of the unfolded protein response and proteosomal overloading. Copper organic acid chelate, the only treatment to show striking and sustained reactive oxygen species generation, had the greatest impact on ubiquitinated proteins, reduced autophagy, and increased aggresome formation, reducing growth in both cell lines. The least effect was noted in copper proteinate with negligible impact on aggresome formation or extended growth for either cell line.

**Conclusion:**

The type and source of copper can impact significantly at the cellular level.

## 1 INTRODUCTION

Copper is perhaps the most regulated heavy metal (Linder & Hazegh‐Azam, 1996), resulting in little toxicity or deficiency. Exceptions of toxicity include Wilson's disease (defective excretion due to ATP7B mutation), and of deficiency include Menkes disease (defective absorption due to ATP7A mutation). Too much or too little can be detrimental on health, and detecting these changes is problematic as existing biomarkers are generally only sensitive enough to detect severe cases (Bost et al., 2016). Yet, copper supplementation is popular, increasing iron and energy levels in humans, and for growth promotion and reducing infection in farm animals.

At the cellular level, the electron transfer potential of copper is important in redox reactions (Nevitt, Ohrvik, & Thiele, 2012).

Copper cellular toxicity is mostly related to the capacity to generate reactive oxygen species (ROS) resulting in oxidative damage to proteins, lipids, and DNA (Gaetke, Chow‐Johnson, & Chow, 2014). For this reason, cellular copper levels are strictly maintained (Nevitt et al., 2012). ROS in cells, normally a result of electron leakage from mitochondria, are involved in many cellular signaling pathways, but increased levels result in oxidative stress and inflammatory responses (Shao et al., 2018; Uriu‐Adams & Keen, 2005) with significant cross talk in transcription factors controlling these responses, namely nrf2 and NF‐κB (Wardyn, Ponsford, & Sanderson, 2015).

Over the last two decades, organic mineral chelates have been increasingly utilized as a trace mineral supply to improve poultry and livestock productivity (Mondal, Paul, Bairagi, Pakhira, & Biswas, 2008; Nollet, Van der Klis, Lensing, & Spring, 2007). Replacing inorganic trace mineral sources with lower levels of more bioavailable organic trace mineral forms not only minimizes the amount excreted but also optimizes trace mineral‐associated performance traits (Abdallah, El‐Husseiny, & Abdel‐Latif, 2009). Typically, organic mineral chelates contain trace metals complexed to either organic acid, amino acid, or peptide ligands, of which several different types of mineral complexes are commercially available. Despite extensive use in agriculture and use as a supplement for humans, a basic understanding of the differences at the cellular level resultant from their use is very limited.

In this study, we analyze the effects of four different sources of copper on proliferating Caco‐2 and HT29 cells to evaluate their effect on cell growth, ROS production, cellular uptake, and effects on protein expression. Three organically complexed sources of copper (copper glycinate—Cu Gly, copper organic acid chelate—Cu OAC, and copper proteinate—Cu Pro) were compared to copper sulfate (CuSO_4_).

## 2 MATERIALS AND METHODS

### 2.1 Chemicals

All chemicals (unless otherwise stated) were obtained from Sigma (Poole, UK). FBS was obtained from Invitrogen. All copper compounds were commercially sourced from independent suppliers.

### 2.2 Cell lines

The human colon carcinoma cell line Caco‐2 (cat. HTB37) was obtained from the American Type Culture Collection and HT29 (cat. 91072201) was obtained from Public Health England Culture Collection. HT29 and Caco‐2 were maintained in MEM supplemented with 1% l‐glutamine and 5% or 10% FBS, respectively (growth medium), under normal conditions (37°C, 5% CO_2_). Both cell lines were mycoplasma negative. These two cell lines were chosen as Caco‐2 is a colon‐derived cell line that, when differentiates, resembles intestinal enterocytes. HT29, also a colon‐derived cell line, resembles goblet cells.

### 2.3 Treatments

To investigate the effect of copper sources, a slightly toxic concentration of IC20 (to reduce viable cell counts by 20%) over a 2‐hr exposure (to reflect the transit in the small intestine, Worsøe et al., 2011) and 10‐hr recovery was chosen. An average IC20 value of 0.4 and 0.5 mM copper source, respectively, for Caco‐2 and HT29 was measured by viable cell counts with trypan blue following the 10‐hr recovery. For all treatments, cells were exposed to freshly prepared 0.4 and 0.5 mM copper solutions in 1% FCS, respectively, for Caco‐2 and HT29 for 2 hr and either assayed directly or allowed to recover in growth medium without added copper for specified periods before analysis.

Reactive oxygen species production was monitored with DCFDA (2′,7′‐dichlorodihydrofluorescein diacetate, Sigma cat 28850). After overnight attachment, cells loaded with DCFDA were exposed to copper solutions for 2 hr. Following washing, fluorescence was measured on a BioTek plate reader (Synergy HT) with excitation at 485 nm and emission at 528 nm. For the 10‐hr recovery, the cells were allowed to recover for just under 8 hr, loaded with DCFDA, and incubated until the 10‐hr recovery point, before measuring fluorescence. Cells were trypsinized and viable cell counts made to obtain ROS generation per cell.

For proteomic analysis and validation by Western blotting, exponentially growing cells were exposed as above to copper compounds for 2 hr and then allowed to recover for 10 hr in growth medium. Cells were washed in PBS, lysed in 2D lysis buffer (7 M urea, 2 M thiourea, 4% CHAPS, and 30 mM Tris, pH 8.0), and quantified by a modified Bio‐Rad assay. Lysates were prepared for mass spectrometry using ReadyPrep 2‐D cleanup kit (Cat 163‐2130; Bio‐Rad) according to the manufacturers’ instructions.

For recovery assays, cells were set up overnight and treated for 2 hr as above, washed twice in fresh medium (without added copper), and allowed to recover for 24, 48, and 72 hr in growth medium, at which point cells were trypsinized and counted.

### 2.4 Measurement of copper uptake by ICP‐MS

Cells were set up as in treatments with two flasks per condition (one for ICP and the other for cell counts) for 2 hr and washed 2 ×  in ice‐cold PBS. The contents of one flask was lysed in 0.4 ml 2D lysis buffer, the copper content was determined by LC‐ICP‐MS (liquid chromatography‐inductively coupled plasma mass spectrometry), and the other flask was trypsinized for cell counts. Samples for LC‐ICP‐MS were digested in HNO_3_ and analyzed using Agilent Technologies 7700 Series ICP‐MS system. Helium gas flow rate used was 4.5 L/min, while the carrier gas was set to 0.81 L/min. Quantitative analysis results were obtained from the MassHunter Workstation Software version A.01.02.

### 2.5 Proteomic analysis

Label‐free LC‐MS/MS was carried out on an Ultimate 3000 nanoLC system coupled to an LTQ Orbitrap XL mass spectrometer as previously described (Meleady et al., 2012). Peptide identification through Proteome Discoverer 2.1 using the search algorithms MASCOT (version 2.3) and SEQUEST HT was used to search against the UniProtKB‐SwissProt fasta database with 20 274 proteins (taxonomy: *Homo sapien)* using the following parameters: (a) MS/MS mass tolerance set at 0.5 Da, (b) peptide mass tolerance set to 20 ppm, (c) carbamidomethylation set as a fixed modification, (d) up to two missed cleavages were allowed, and (e) methionine oxidation set as a variable modification. For further analysis, only peptides with ion scores of 40 and above or XCorr of >1.9 for +1, >2.2 for +2, and >3.75 for +3 charged molecules were chosen (Linge et al., 2014). The following criteria were applied to assign proteins as confidently identified: (a) an ANOVA score between the experimental group of ≤0.05 and (b) a fold change ≥1.5. Venn diagrams were obtained using the Venny software (Oliveros, 2007).

### 2.6 Western blotting

Cell lysates were separated on a 12% or 4%–12% SDS gels and transferred to PVDF, blocked for 2 hr, and then exposed to primary antibodies (Supporting Information Table S1) overnight at 4°C. Secondary antibodies conjugated to horseradish peroxidase were detected by enhanced chemiluminescence (Luminol, Santa Cruz, CA, USA). Densitometry was carried out using TotalLab software, TL100.

### 2.7 Autophagy detection assay

The Cyto‐ID Autophagy detection kit (Enzo ENZ‐51031) was used to evaluate autophagy following manufacturer's instructions. Caco‐2 cells and HT29 cells were exposed to copper solutions for 2 hr followed by a 10‐hr recovery in growth medium. Exposure to 30 μM chloroquine in 1% FCS was used as a positive control. The CYTO‐ID detection reagent (detects autolysosomes and early autophagic vacuoles) was measured at 480 nm excitation and 530 nm emission, and was normalized to the nuclear Hoechst 33342 (340 nm excitation and 480 nm emission) to give a mean fluorescent intensity per cell that was then compared to control cells not exposed to copper compounds.

### 2.8 Aggresome detection

The ProteoStat Aggresome detection kit (Cat ENZ‐51035) was used to detect denatured protein cargo found within aggresomes following manufacturer's instructions. Caco‐2 and HT29 were set up on glass slides and were exposed to copper solutions for 2 hr followed by a 10‐hr recovery and a 24‐hr recovery. MG132 was used as a positive control incubated for 10 hr or 24 hr. Hoechst 33342 and Aggresome detection reagent were imaged with DAPI and Texas Red filter sets at 20 ×  magnification and images superimposed using MetaMorph (version 7.7.70).

### 2.9 Aggregate detection

Protein aggregates were determined by fractioning cell lysates into soluble (Triton X‐100) and insoluble fractions that contain aggregates (SDS) as previously described (Rapino, Jung, & Fulda, 2014). Cell lysates taken 10 and 24 hr postrecovery for Caco‐2 and HT29 cells, respectively, were lysed in Triton X‐100 (2% in PBS with protease and phosphatase inhibitors) and incubated on ice for 30 min, before centrifuging at 18,800 *g* for 30 min at 4°C. The soluble fraction (supernatant) and the insoluble fraction (resuspended in 1% SDS) were analyzed for ubiquitin‐positive proteins by Western blotting.

### 2.10 Statistics

Statistics were carried out using the Student's *t* test assuming a two‐tailed distribution and two samples of unequal variance. Values of *p *<* *0.05 were considered as statistically significant.

## 3 RESULTS

### 3.1 ROS production

**Figure 1:**
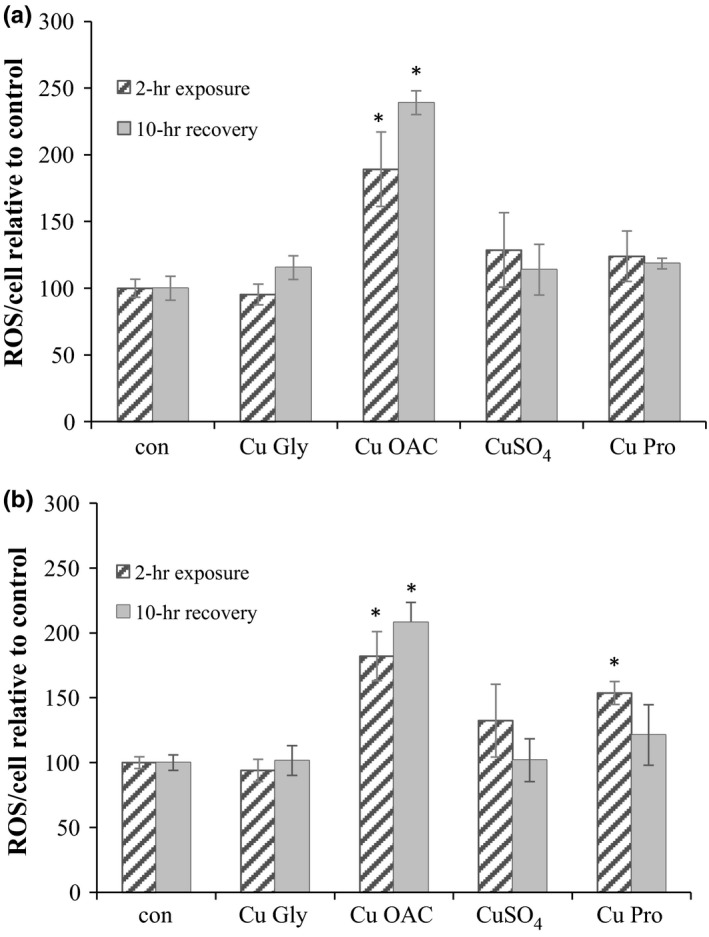
ROS production in HT29 (a) and Caco‐2 (b) after 2‐hr exposure and 10‐hr recovery. Results are the average of at least three separate repeats. * denotes statistical significance of *p* < 0.05 compared to the control for either 2‐hr exposure or 10‐hr recovery. ROS, reactive oxygen species

Reactive oxygen species generation in Caco‐2 and HT29 cells was measured after a 2‐hr exposure and also after a 10‐hr recovery phase. The most striking result was with Cu OAC whereby ROS increased almost twofold in HT29 and Caco‐2 cells (*p* = 0.036 and *p* = 0.001, respectively) compared to control after 2‐hr exposure (Figure 1) and was maintained following the 10‐hr recovery phase with a 2.4‐fold increase noted in HT29 (*p* = 0.001) and 2.1‐fold increase in Caco‐2 (*p* = 0.003) cells noted relative to the control. CuSO_4_ and Cu Pro caused transient ROS generation in Caco‐2, being reduced and not significant following the 10‐hr recovery. The ligands used in the manufacture of the organic copper complexes did not affect ROS production (data not shown).

### 3.2 Copper uptake on 2‐hr exposure

**Figure 2:**
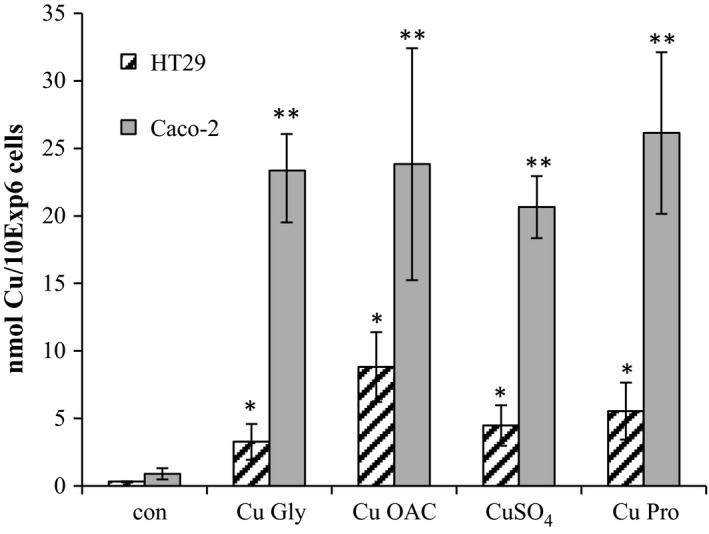
Copper uptake after 2‐hr exposure to copper compounds as measured by LC‐ICP‐MS. Results are the average of three separate repeats. * and **denotes significant differences for HT29 and Caco‐2 cells, respectively, exposed to coppers relative to control (*p* < 0.05)

As the bioavailability of the organic and inorganic copper sources may result in different uptake rates, the intracellular copper levels were measured by LC‐ICP‐MS. Both cell lines took up copper from all compounds (Figure 2) with Caco‐2 displaying a much higher capacity for uptake (2.8‐fold higher than HT29). For Caco‐2, uptake levels were quite similar for all copper compounds tested, perhaps indicating saturation; the lowest was CuSO_4_ with a 22.7‐fold increase (*p* = 0.004), and the highest was with the Cu Pro with a 28.7‐fold increase (*p* = 0.02). For HT29, Cu Gly resulted in the smallest increase in copper uptake (10‐fold relative to control) while Cu OAC had the highest at 26‐fold, *p* = 0.036.

### 3.3 Transporter expression on 2‐hr exposure to copper compounds

**Figure 3:**
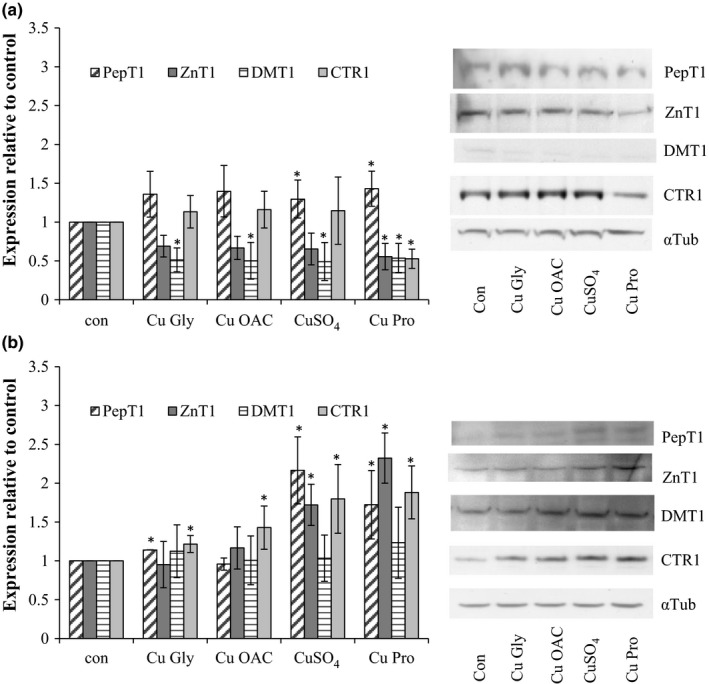
Expression of relevant transporters in HT29 (a) and Caco‐2 (b) cells after 2‐hr exposure to copper compounds. Results are the average of at least three separate repeats. Representative blots are shown

As the organic copper sources have been reported to be more efficiently absorbed than inorganic copper in vivo (European Medicines Agency, 2007), the impact on transporters related to metal or peptide uptake (copper transporter 1—CTR1, zinc efflux transporter 1—ZnT1, di‐metal transporter 1—DMT1, and peptide transporter 1—PepT1) was investigated. In Caco‐2 cells, PepT1, ZnT1, and CTR1 were increased particularly for CuSO_4_ and Cu Pro, and no change in DMT1 (Figure 3). In HT29 cells, all compounds reduced ZnT1 and DMT1 levels, without affecting CTR1 levels. PepT1 was only significantly increased in CuSO_4_ and Cu Pro.

### 3.4 Proteomic analysis of copper‐treated Caco‐2 and HT29 cells after a 10‐hr recovery

**Figure 4:**
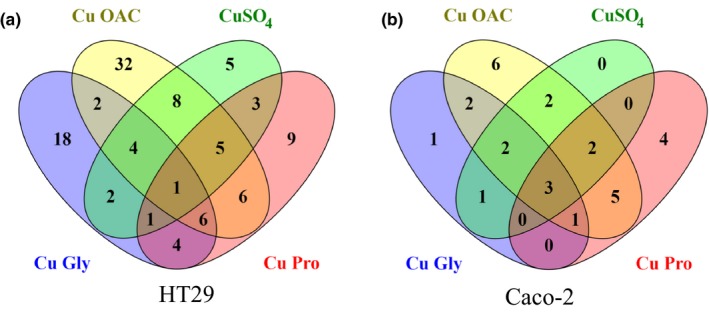
Venn diagrams showing the number of overlaps in differentially expressed proteins in HT29 (a) and Caco‐2 (b) cells. Protein lists were submitted to Venny to generate overlaps

Both cell lines were subjected to proteomic analysis following a 10‐hr recovery phase after the 2‐hr copper treatment (Tables 1, 2, 3, 4). For both cell lines, the highest number of differentially expressed proteins and uniquely expressed proteins was seen with Cu OAC and the least with CuSO_4_ (Figure 4, Supporting Information Tables S2–S8). Metallothionein (MT) was found to be increased in both cell lines (and to be the most highly increased of proteins), as were antioxidant response proteins, heme oxygenase 1 (HMOX1) in Caco‐2 and sulfiredoxin 1 (SRXN1) in HT29 (Tables 1 and 3).

**Table 1:** Increased protein expression in HT29 cells treated with organic or inorganic copper compared to control cells (2‐hr exposure and 10‐hr recovery)

Accession	ANOVA (*p*)	Gene	Description	Cu Gly	Cu OAC	CuSO_4_	Cu Pro
ER/UPR/Proteosomal							
P08240	0.0147	SRPR	Signal recognition particle receptor subunit alpha	*1.7*	1.7	2.0	2.6
P49368	0.0202	CCT3	T‐complex protein 1 subunit gamma	*2.3*	2.4	2.3	*1.8*
P40227	0.0155	CCT6A	T‐complex protein 1 subunit zeta	2.4	2.2	2.2	*2.3*
Trafficking							
P09497	0.0102	CLTB	Clathrin light chain B	*1.6*	1.8	*1.8*	1.8
Q13492	0.0065	PICALM	Phosphatidylinositol‐binding clathrin assembly protein	1.8	2.4	*2.1*	2.4
Cytoskeletal							
Q14008	0.0286	CKAP5	Cytoskeleton‐associated protein 5	*1.4*	1.6	1.5	*1.4*
P50552	0.0130	VASP	Vasodilator‐stimulated phosphoprotein	*2.1*	2.2	2.2	*2.8*
Q14204	0.0005	DYNC1H1	Cytoplasmic dynein 1 heavy chain 1	2.3	3.1	2.5	*2.2*
Apoptosis							
P55957	0.0121	BID	BH3‐interacting domain death agonist	*1.2*	*1.1*	1.6	1.6
Translation							
P13639	0.0004	EEF2	Elongation factor 2	1.6	1.7	1.7	*2.1*
P51991	0.0056	HNRNPA3	Heterogeneous nuclear ribonucleoprotein A3	*1.9*	1.9	*1.6*	1.6
O60832	0.0034	DKC1	H/ACA ribonucleoprotein complex subunit 4	*1.9*	*2.6*	2.0	2.5
Q07020	0.0014	RPL18	60S ribosomal protein L18	*1.7*	2.5	2.2	1.8
P62241	0.0065	RPS8	40S ribosomal protein S8	*3.5*	5.7	5.0	*5.6*
Metabolic							
Q00796	0.0004	SORD	Sorbitol dehydrogenase	2.0	2.2	2.5	2.8
Antioxidant response							
Q9NNW7	0.0213	TXNRD2	Thioredoxin reductase 2, mitochondrial	1.7	*1.3*	*1.7*	1.7
Q16881	0.0077	TXNRD1	Thioredoxin reductase 1, cytoplasmic	*2.0*	2.8	1.8	3.0
Q9BYN0	0.0006	SRXN1	Sulfiredoxin‐1	2.8	7.2	*1.4*	2.4
Q8IYD1	0.0020	ERF3B	Eukaryotic pcrf GTP‐binding subunit	*2.7*	3.9	3.5	*4.6*
C9JRZ8	0.0438	AKR1B15	Aldo‐keto reductase family 1 member B15	1.5	1.8	*1.3*	1.8
Transcription							
Q15459	0.0142	SF3A1	Splicing factor 3A subunit 1	*1.2*	1.5	*1.5*	1.6
Autophagy							
Q13501	0.0001	SQSTM1	Sequestosome‐1	1.5	2.4	*1.2*	1.9
Metal binding							
P04732	0.0017	MT1E	Metallothionein‐1E	13.9	9.3	*8.2*	16.2
P13640	0.0054	MT1G	Metallothionein‐1G	26.8	16.9	*15.0*	26.2

*Note*

Italic entries denote values that were not statistically significant in a paired *t* test with the control cells.

**Table 2:** Decreased protein expression in HT29 cells treated with organic or inorganic copper compared to control cells (2‐hr exposure, 10‐hr recovery)

Accession	ANOVA (*p*)	Gene	Description	Cu Gly	Cu OAC	CuSO_4_	Cu Pro
ER/UPR/Proteosomal							
P13667	0.0102	PDIA4	Protein disulfide‐isomerase A4	*1.3*	1.7	1.8	1.8
Trafficking							
Q92928	0.0185	RAB1C	Putative Ras‐related protein Rab‐1C	130.5	*3.0*	*1.9*	148.5
P02765	0.0332	AHSG	Alpha‐2‐HS‐glycoprotein	1.8	2.6	2.1	2.9
P20073	0.0242	ANXA7	Annexin A7	*1.2*	*1.4*	1.5	1.6
Cytoskeletal							
Q15424	0.0072	SAFB	Scaffold attachment factor B1	468.3	*2.5*	*1.7*	565.7
P05787	0.0242	KRT8	Keratin, type II cytoskeletal 8	1.8	2.0	*1.2*	*1.4*
P60709	0.0387	ACTB	Actin, cytoplasmic 1	*1.4*	1.6	1.7	*1.8*
P98088	0.0067	MUC5AC	Mucin‐5AC	1.9	2.3	*1.8*	*2.0*
Apoptosis							
Q16891	0.0325	IMMT	MICOS complex subunit MIC60	*1.4*	1.8	1.5	*1.3*
Translation							
P19338	0.0057	NCL	Nucleolin	1.7	*1.0*	1.7	1.9
P55884	0.0191	EIF3B	Eukaryotic translation initiation factor 3B	*1.3*	1.6	*1.6*	1.8
P24534	0.0202	EEF1B2	Elongation factor 1‐beta	*1.3*	1.5	1.4	1.6
O00461	0.0021	GOLIM4	Golgi integral membrane protein 4	2.1	*1.29* a	1.5	*1.4*
Metabolic							
P09622	0.0380	DLD	Dihydrolipoyl dehydrogenase, mitochondrial	*1.5*	1.7	1.7	1.9
P37837	0.0219	TALDO1	Transaldolase	*1.3*	1.5	1.5	1.7
Cell cycle							
P62714	0.0026	PPP2CB	S/t‐protein phosphatase 2A catalytic subunit beta isoform	2.3	*1.89* a	1.8	1.4
A2A2Z9	0.0438	ANKRD18B	Ankyrin repeat domain‐containing protein 18B	1.6	1.5	1.9	*1.6*

*Note*

Italic entries denote values that were not statistically significant in a paired *t* test with the control cells.

Values that are increased compared to control, while the rest of the results for that protein are decreased compared to control.

**Table 3:** Increased protein expression in Caco‐2 cells treated with organic or inorganic copper compared to control cells (2‐hr exposure and 10‐hr recovery) with ANOVA value of *p* < 0.05

Accession	ANOVA (*p*)	Gene name	Description	Cu Gly	Cu OAC	CuSO_4_	Cu Pro
ER/UPR/Proteosomal							
Q92598	5.0E‐05	HSPH1	Heat‐shock protein 105 kDa	1.5	2.5	1.6	2.0
P17066	1.0E‐05	HSPA6	Heat‐shock 70 kDa protein 6	2.5	6.2	*2.8*	2.6
P25685	1.0E‐04	DNAJB1	DNAJ homolog subfamily B member 1	*2.3*	5.8	*2.6*	3.5
P62979	0.04	RPS27A	Ubiquitin‐40S ribosomal protein S27a	*1.3*	1.8	1.5	*1.2*
Trafficking/signaling							
O94907	0.002414	DKK1	Dickkopf‐related protein 1	*1.71*	2.35	*2.54*	3.51
Cytoskeletal							
Q14315	2.7E‐05	FLNC	Filamin‐C	1.5	1.9	*1.4*	2.2
Q5BJD5	0.046	TMEM41B	Transmembrane protein 41B	1.6	1.9	*1.5*	*1.7*
Q9UHB6	0.0281	LIMA1	LIM domain and actin‐binding protein 1	*1.2*	1.6	1.4	1.5
Metabolic							
P51857	0.0047	AKR1D1	3‐oxo‐5‐beta‐steroid 4‐dehydrogenase	1.8	1.7	1.8	2.2
Q01581	0.0017	HMGCS1	Hydroxymethylglutaryl‐CoA synthase, cytoplasmic	*1.4*	1.6	*1.3*	1.8
Antioxidant response							
P09601	6.5E‐07	HMOX1	Heme oxygenase 1	15.6	24.2	14.6	12.4
P07602	0.0012	PSAP	Prosaposin	1.6	1.4	1.5	*1.0*
Transcription							
P54727	0.0117	RAD23B	UV excision repair protein RAD23 homolog B	*1.6*	2.8	1.6	*1.2*
Autophagy							
Q13501	0.0003	SQSTM1	Sequestosome‐1	2.7	3.6	2.8	*2.2*
O95817	0.0006	BAG3	BAG family molecular chaperone regulator 3	4.3	8.3	*3.9*	*4.4*
Metal binding							
P02795	3.3E‐07	MT2A	Metallothionein‐2	*5.3*	8.0	13.5	79.5
P04733	2.7E‐07	MT1F	Metallothionein‐1F	*24.9*	33.2	75.3	206.5
Q06481	0.006	APLP2	Amyloid‐like protein 2	1.7	1.8	1.7	*1.0*

*Note*

Italic entries denote values that were not statistically significant in a paired *t* test with the control cells.

**Table 4:** Decreased protein expression in Caco‐2 cells treated with organic or inorganic copper compared to control cells (2‐hr exposure and 10‐hr recovery) with ANOVA value of *p* < 0.05

Accession	ANOVA (*p*)	Gene name	Description	Cu Gly	Cu OAC	CuSO_4_	Cu Pro
Cytoskeletal							
P35611	0.0012	ADD1	Alpha‐adducin	0.8	0.5	*0.9*	0.6

*Note*

Italic entries denote values that were not statistically significant in a paired *t* test with the control cells.

The impact of the different copper sources on protein expression (Figure 4, Tables 1, 2, 3, 4) showed only one protein, sorbitol dehydrogenase (SORD), to be differentially expressed in HT29, with highest expression in Cu Pro‐treated cells. For Caco‐2, there were three shared proteins, HMOX1, HSPH1 ad AKR1D1. Highest AKR1D1 expression was noted in Cu Pro, while HMOX1 and HSPH1 were highest in Cu OAC‐treated cells.

Comparing the organic copper sources to the inorganic form in HT29, six proteins were shared between the organic compounds that were significantly altered compared to the control, and were not altered significantly in CuSO_4_‐treated cells (Figure 4a and Table 1). These included metal binding proteins (MT1E and MT1G), redox‐related proteins (aldo‐keto reductase family 1 member B15 (AKR1B15) and SRXN1), and autophagy‐related proteins (sequestosome‐1 (SQSTM1) and phosphatidylinositol‐binding clathrin assembly protein (PICALM)).

For Caco‐2, comparison of the organic to the inorganic copper sources indicated that only one protein was differentially expressed in the organic copper compounds, heat‐shock 70 kDa protein 6 (HSPA6) with highest expression in Cu OAC at 6.2‐fold compared to the control (Figure 4b).

**Figure 5:**
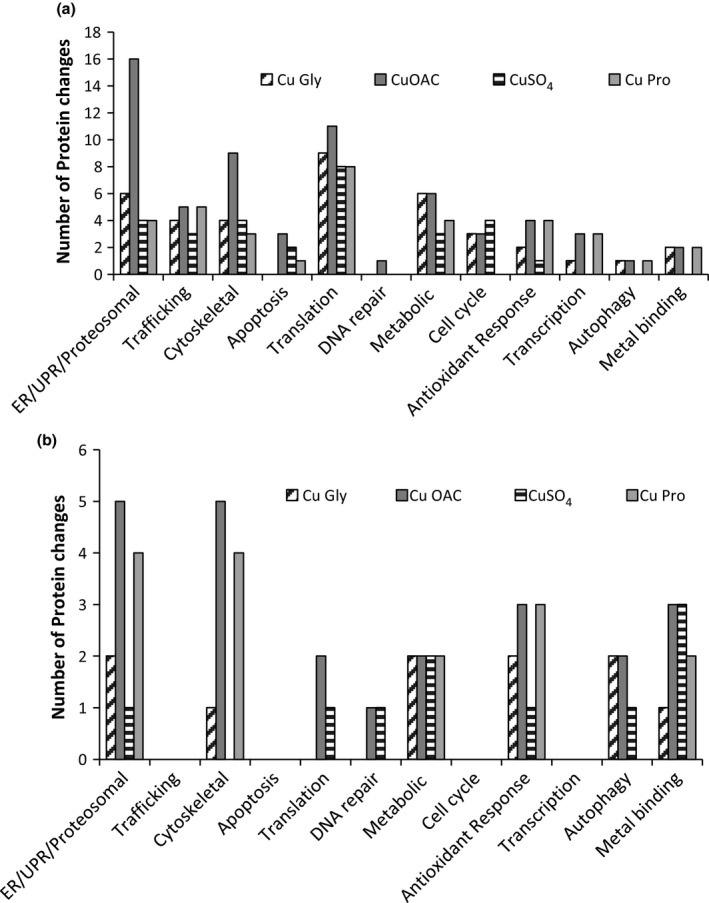
Biological function of differentially expressed proteins in copper‐treated HT29 (a) and Caco‐2 cells (b). Results are shown as the number of differentially expressed proteins sharing biological function as determined by PubMed

Biological processes most affected by treatment included protein translation, ER function, and the unfolded protein response (Figure 5, Tables 1, 2, 3). In HT29, organic copper sources showed greater changes in proteins involved in antioxidant response, transcription, autophagy, and metal binding observed than inorganic copper, which only caused a marginal increase in proteins involved in cell cycle. In Caco‐2, inorganic copper supplementation produced less changes in cytoskeletal, ER/UPR/Proteosomal, and antioxidant responses than the organic copper compounds.

### 3.5 Validation of proteomic targets

**Figure 6:**
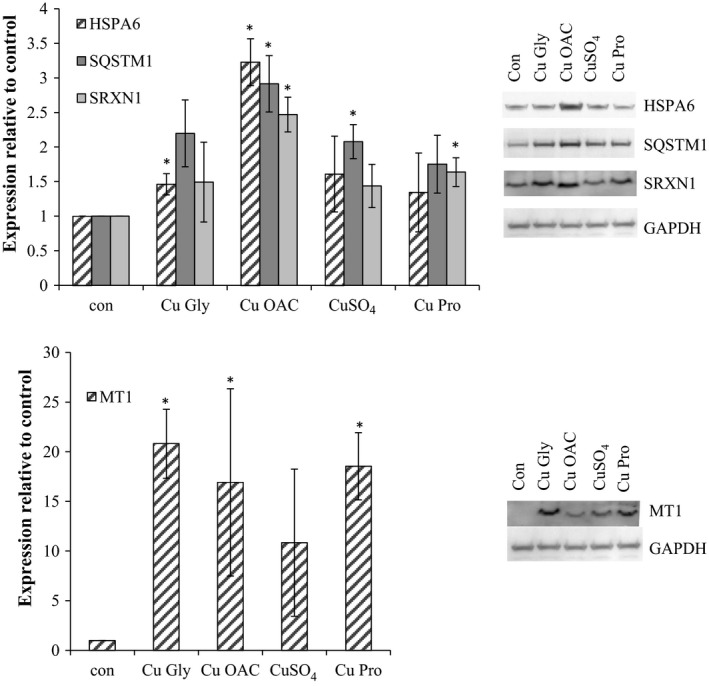
Validation of HT29 proteomic target proteins HSPA6, SQSTM1, SRXN1, and MT1. Results are the average of at least three separate repeats. Representative blots are shown. Asterisks indicate *p* < 0.05 when compared to the control

**Figure 7:**
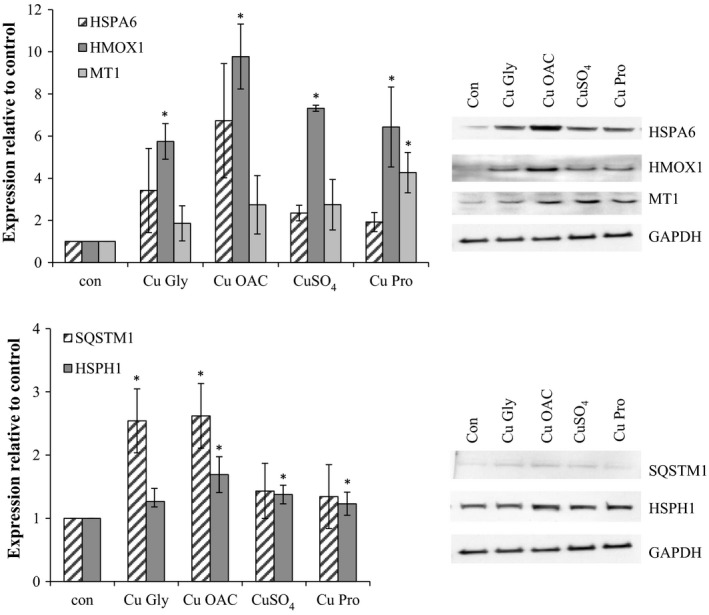
Validation of Caco‐2 proteomic target proteins HSPA6, HMOX1, MT1, SQSTM1, and HSPH1, with GAPDH as the loading control. Results are the average of at least three separate repeats. Representative blots are shown. Asterisks indicate *p* < 0.05 when compared to the control

Targets from proteomic analysis were assessed by Western blotting. For HT29, MT1, SRXN1, SQSTM1, and HSPA6 were selected and validated as representative of biological functional groups (Figure 6). HMOX1, HSPH1, HSPA6, MT1, and SQSTM1 were chosen for Caco‐2 cells, representing different groups of biological function, and were validated, although the fold change in MT1 expression in Western blots was lower than that observed in proteomic analysis (Figure 7, Table 3).

### 3.6 Impact of copper exposure on protein misfolding

**Figure 8:**
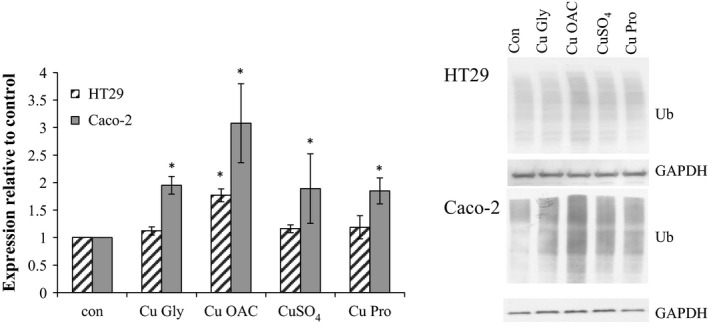
Expression of high molecular weight ubiquitinated proteins in HT29 and Caco‐2 cells with GAPDH as the loading control. Results are the average of at least three separate repeats. Representative blots are shown. Asterisks indicate *p* < 0.05 when compared to the control

Proteomic analysis pointed to changes in proteosomal proteins and increased expression of protein chaperones involved in handling misfolded proteins. The presence of high molecular weight ubiquitinated proteins was indicative of overloaded proteosomal degradation (Figure 8) with Cu OAC causing the greatest effect. While modest in HT29 cells (1.8‐fold), the effect was more pronounced in Caco‐2 with a threefold increase compared to the control (p < 0.05).

### 3.7 Autophagy

**Figure 9:**
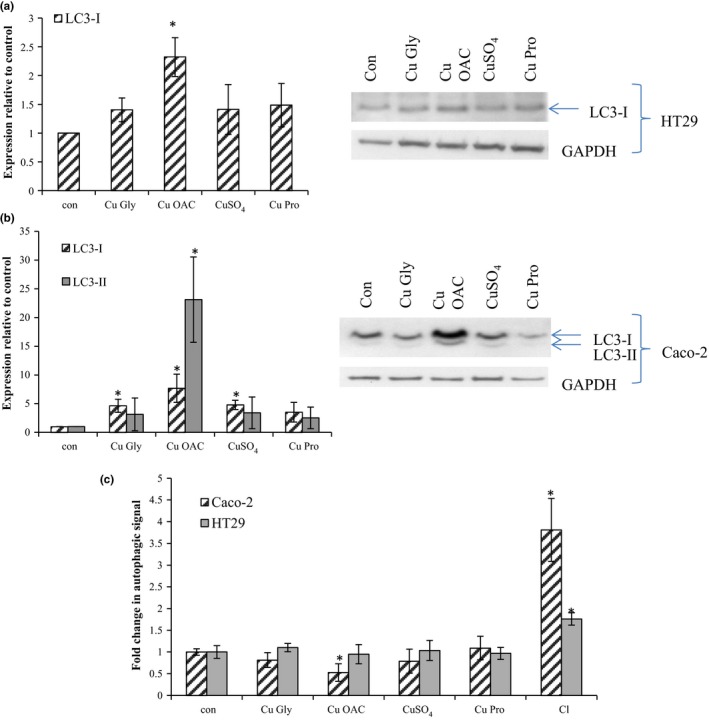
Expression of autophagy marker LC3‐I and LC3‐II in HT29 (a) and Caco‐2 (b) cells with GAPDH as the loading control. Results are the average of at least three separate repeats. Representative blots are shown. (c) Autophagic signal in Caco‐2 and HT29 cells. Results are expressed as the fold change in autophagic signal (FITC), normalized initially to DAPI and then to the control. Chloroquine (Cl) with 1% serum was used as a positive control. Results are the average of at least three separate repeats. Asterisks indicate *p* < 0.05 when compared to control

Increased SQSTM1 an autophagy adaptor protein, seen in both cell lines, may be due to oxidative stress or stalling in autophagic flux. Microtubule‐associated protein 1 light chain 3 (LC3‐I) protein, whose increased expression or conversion to LC3‐II are important markers of autophagy, was increased by 2.3‐fold compared to control in HT29 without conversion to LC3‐II (Figure 9a). For Caco‐2, expression of LC3‐I and LC3‐II was increased following exposure to Cu OAC by 7.7‐fold and 23‐fold, respectively (Figure 9b). These may indicate increased autophagy or stalling of autophagy at the 10‐hr recovery point; however, a fluorescent autophagy assay showed no increase in fluorescence in HT29 or Caco‐2 cells treated with Cu OAC (Figure 9c) compared to control. In fact, the only significant result was a reduction in autophagy in Caco‐2 on treatment with Cu OAC (47% reduction, p = 0.018) when compared to the control cells.

### 3.8 Aggresome formation

**Figure 10:**
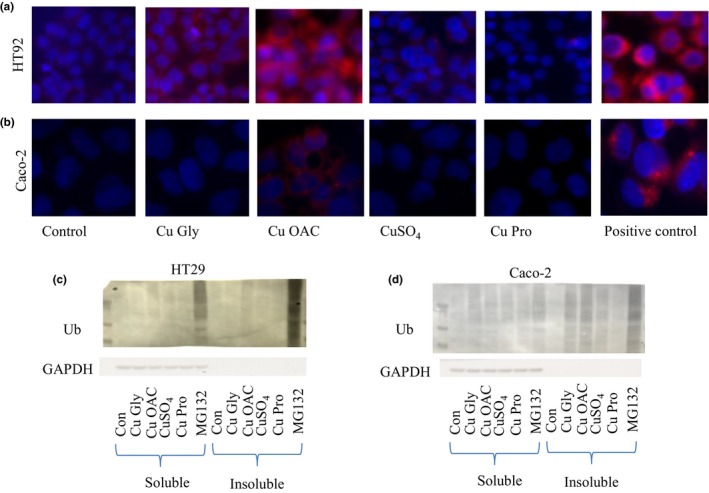
Fluorescent aggresome images of HT29 (a) and Caco‐2 (b) after a 2‐hr exposure and 24‐ and 10‐hr recovery, respectively. The aggresome detection reagent shown in red is present close to nucleus (stained with Hoechst). Images were combined with MetaMorph and are representative of at least five replicates for each cell line. MG132 was used as a positive control that inhibits proteosomal activity. (c) and (d) show the presence of ubiquitinated proteins in HT29 and Caco‐2 cells, respectively, in the soluble and insoluble fraction (contains aggregated proteins). MG132 shows strong staining especially in the insoluble fraction. GAPDH was used a loading control, and the lack of signal in the insoluble fraction indicates good separation

Aggresomes are inclusion bodies of misfolded proteins located in a perinuclear space that serve to protect cells from the damaging effects of aggregates, and LC3 and SQSTM1 expression have been linked with aggresome formation. Using a fluorescent detection kit, aggresomes were detected in Cu OAC‐treated HT29 and Caco‐2 cells (Figure 10a and b). Cu Pro‐treated cells did not display signs of aggresome formation in either cell line, while Cu Gly and CuSO_4_ caused some sporadic aggresome formation in HT29 but not in Caco‐2. Isolation of aggresomes through sequential solubilization in Triton X‐100 and then in SDS demonstrated the presence of ubiquitinated protein aggregates (typical of aggresome formation) following Cu OAC addition and to a lesser extent in Cu Gly‐ and CuSO_4_‐supplemented cells (Figure 10c and d). Cu Pro addition resulted in the lowest level of ubiquitinated proteins in the insoluble fraction of the four copper sources tested.

### 3.9 Impact of copper treatment on cellular survival

**Figure 11:**
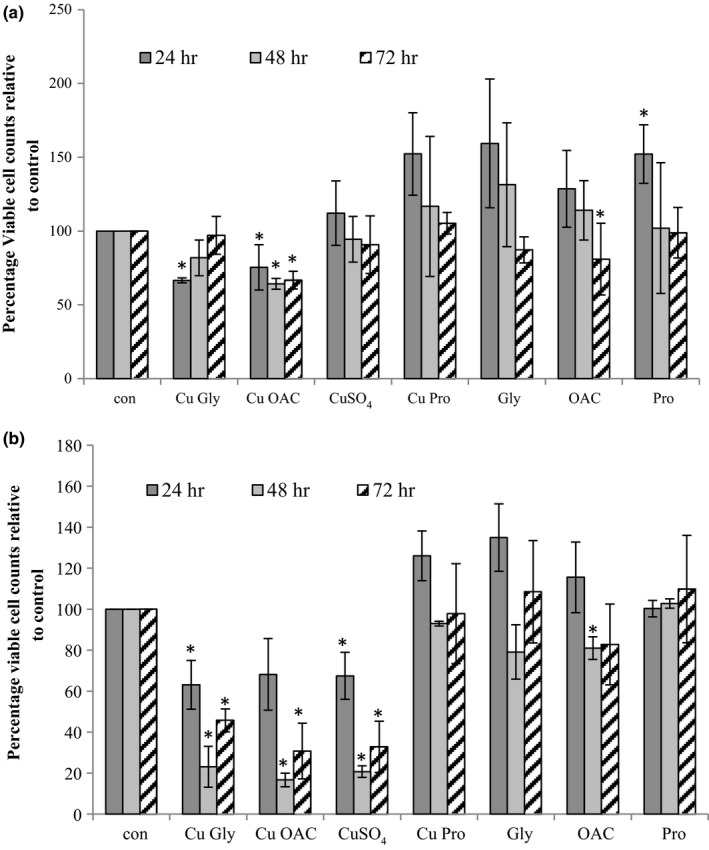
Long‐term cellular growth response after copper treatment. Cells exposed to copper compounds for 2 hr, were allowed to recover, and trypsinized after 24‐, 48‐ and 72‐hr recovery. Total and viable cell counts were made and expressed relative to the control. The results are expressed as the average of at least three replicates. Asterisks indicate *p* < 0.05 compared to the control

With the presence of many ubiquitinated high molecular weight proteins, the impact of the 2‐hr copper treatment on cells beyond the 10‐hr recovery was assessed (Figure 11). In HT29, neither CuSO_4_ nor Cu Pro had a lasting effect on growth over the 72 hrs monitored. Cu Gly caused a 33% reduction in growth (p = 0.04) over the initial 24 hrs but did not affect growth thereafter. Cu OAC significantly reduced growth over the three time points monitored, effecting a 33% reduction even after 72‐hr recovery (p = 0.01). In Caco‐2, the impact on cell growth was more severe. Both Cu Gly and Cu OAC showed similar reductions in growth to CuSO_4_ that were compounded with time reaching 46%, 31%, and 32% of the control, respectively. Cu Pro was the only copper treatment that did not negatively impact on the growth of the cells.

## 4 DISCUSSION

Copper is an essential micronutrient, being involved in many biological processes from photosynthesis to aerobic respiration (Nevitt et al., 2012). In humans, copper is the most tightly controlled micronutrient (Gaetke et al., 2014) through absorption/excretion, although intestinal regulation is poorly understood (Uriu‐Adams & Keen, 2005). Daily intake ranges from 1 to 2 mg with upper allowable levels of 10 mg per day have been recommended (European Medicines Agency (EMEA), 2007) for individuals with normal copper homeostasis. Intake is normally achieved in European countries through diet (Flynn et al., 2009), while, in the United States, supplements contribute significantly to intake (Bailey, Fulgoni, Keast, & Dwyer, 2011). For animal feed, the concentrations depend on the species, with current European maximum copper levels ranging from 15 to 35 mg/Kg feed for most farm animals and reaching as high as 50 mg/Kg for crustaceans (EFSA Panel on Additives and Products or Substances used in Animal Feed (FEEDAP), 2016).

Copper toxicity can result in vomiting, diarrhea, hemolytic anemia, gastrointestinal bleeding, and multiple target organ failure (liver and kidneys), and is normally as a result of intake of contaminated water or contaminated foodstuffs, mostly as a result of industrial, domestic, and agricultural activities (Ali & Al‐Qahtani, 2012; Alturiqi & Albedair, 2012). Geographical location, hardness of water, and pH as well as the presence of copper piping (US National Academy of Science, 2000) affect increased copper levels, impacting on commercially important stocks of fish and crustaceans, as well as their biodiversity (Solomon, 2009). As copper sediments in water after interacting with organic matter and settles on the seabed, crustaceans and shellfish such as shrimp, crabs, prawns, and oysters are being exposed to higher levels of copper and may exceed upper levels suitable for consumption (Abdel‐Salam & Hamdi, 2014). Overdosing on supplements has also been reported. For human supplements, most are sold as 2 mg copper per tablet (one‐a‐day tablet). In one case, an individual who took 30 mg per day for two years and then 60 mg per day for almost a year had extensive liver damage and required a liver transplant (O'Donohue, Reid, Varghese, Portmann, & Williams, 1999).

At the cellular level, it is the transition state of copper (oxidized and reduced) that, while essential in many redox reactions, can result in the generation of reactive oxygen/nitrogen species that, at high concentrations, damage proteins, lipids, and DNA (Gaetke et al., 2014). In this study, two colon cancer cell lines, Caco‐2 and HT29, were selected (because of their similarity to normal intestinal enterocyte and goblet cells) to examine ROS production on exposure to inorganic CuSO_4_ and organic nutrient sources of copper (Cu Gly, Cu OAC, and Cu pro) and their global proteomic impact. Employing a two‐hour exposure to reflect the average intestinal transit (Worsøe et al., 2011), a concentration of copper which caused a small but significant impact on cell viability was chosen. For the four coppers, the average concentrations were 400 μM and 500 μM for Caco‐2 and HT29, respectively, which caused a 30% reduction in cell growth. Comparing these concentrations to levels in supplements and food additives is not straightforward for humans as much of the information in the literature is based on mg Copper per day, and is complicated by the presence of other foodstuffs, metals, or phylates that can interfere with absorption (Araya et al., 2006; Jacela et al., 2010). Guidelines for the no‐observable‐adverse‐effect level (NOAEL) in drinking water was 6 mg/L (94.4 μM) (WHO/SDE/WSH/03.04/88, 2004) and this concentration was employed by Baurely et al. (2003) when looking at the response of suckling rat pups and differentiated Caco‐2 to copper (3 and 94 μM)(Bauerly, Kelleher, & Lonnerdal, 2005). In acute copper exposure, concentrations in excess of 30 mg/l (472 μM) caused illness (Centers for Disease Control and Prevention (CDC), 1996) while another study found 4 to 70 mg/L (62 and 1101 μM) caused illness (US National Academy of Science, 2000). Such differences between and even within studies may reflect variations in sensitivity to copper within normal populations (Elliot, Frio, & Jarman, 2017).

Of the copper compounds tested, Cu OAC had the most striking effect on both cell lines, causing significant sustained ROS, while only a transient increase was noted with Cu Pro. Cellular copper uptake levels reflected ROS in HT29 but not in Caco‐2. As proteins are one of the main targets for ROS damage, the impact of the copper compounds was assessed via proteomics.

Proteomic expression profiling after 10‐hr copper recovery showed further differences between the copper sources. As expected, there were shared changes in proteins involved in oxidative stress and copper storage but there were also copper source‐specific and uniquely altered protein expression patterns noted. It is perhaps no coincidence that Cu OAC caused the greatest proteomic changes given the sustained ROS generation. It should be noted that as only one time point was analyzed, it is possible that proteins involved in very early response may have been missed. Indeed, the changes in the transporters detected at the end of the two‐hour exposure were not detected after the 10‐hour recovery.

Shared protein changes involved in oxidative stress were sorbitol dehydrogenase (SORD) in HT29 and HMOX1 in Caco‐2. SORD is a component of the polyol pathway and increased activity resulted in increased peroxynitrite and reduced glutathione (GSH) (Tang et al., 2010). Heme oxygenase 1 (HMOX1) is essential in heme catabolism, and increased expression is induced through nrf2 signaling during oxidative stress (Wang et al., 2012) and has also been implicated in lipid peroxidation (Peña, Colbenz, & Kiselyov, 2015). While HMOX1 expression was increased in all copper sources in Caco‐2 cells, this did not reflect the ROS levels as detected by DCFDA. Metal storage or detoxification by metallothionein (MT) expression was observed in both cell lines, being the most increased protein both cell lines. Metallothionein is a small protein (6‐7 kDa) with up to nine copper binding sites that allows it to mop up excess metals especially zinc and copper.

Reactive oxygen species generation and oxidative stress are also implicated in pro‐inflammatory signaling cascades with extensive cross talk between NF‐ĸB and the antioxidant response transcription factor nrf2 reported (Wardyn et al., 2015). In this study, increased expression of HMOX1 and Sequestosome‐1 suggest activated nrf2 signaling. Activation of NF‐κB results in the secretion of inflammatory signals (IL‐6, IL‐8, and PGE2), as well as activation of ERK/JNK signaling and changes in expression of a variety of proteins (Morgan & Liu, 2011). In this study, while whole cell lysates at a 10‐hour recovery would not be suited to detecting changes in secreted proteins or detecting changes in phosphorylation of ERK/JNK, changes in levels of antioxidant targets of NF‐κB including superoxide dismutase (1 and 2), ferritin heavy chain, GST‐pi, AkR1C1, and inducible nitric oxide synthase were not detected. Timing may be critical for detection of inflammatory responses, secreted Il‐6, Il‐8, and PGE2 protein have been detected in Caco‐2 and HT29 supernatant at 24 hrs, while mRNA can be detected as early as six‐hour exposure to inflammatory signals (Kim et al., 2009; Van De Walle, Hendrickx, Romier, Larondelle, & Schneider, 2010), whereas antioxidant proteins such as HMOX1 can be detected as early as four hours in macrophages (Alcaraz et al., 2004).

Protein changes specific to organic copper sources included six proteins that were significantly differentially expressed in HT29, with only one protein, HSPA6, noted in Caco‐2 and previously detected in response to proteosomal stress (Noonan, Gregory Fournier, & Hightower, 2008). In HT29, SQSTM1, AKR1B15, PICALM, SRXN1, and MT1E and MT1G were only significantly altered following treatment with the organic copper compounds. SRXN1 is a redox‐activated thiol switch important in the reversal of glutathionylation reactions (Findlay et al., 2006), while AKR1B1 is involved in lipid peroxidation detoxification (Barski, Tipparaju, & Bhatnagar, 2008). SQSTM1 and PICALM have both been implicated in autophagy (Moreau et al., 2014; Pankiv et al., 2007). However, given the small differences between the organic copper compounds and CuSO_4_ and the very variable response with the inorganic copper product, these may not be suitable markers to monitor organic copper overload.

Some of the greatest changes in proteins involved endoplasmic reticulum function, unfolded protein response (UPR), and proteosomal activity, and this was further noted by increases in high molecular weight ubiquitinated proteins particularly in Cu OAC (Figure 8).

Proteosomal overload leads to increased amounts of ubiquitinated misfolded proteins that interact with each other to form aggregates causing proteotoxicity. To alleviate this, aggregates can be degraded by autophagy (Lamark & Johansen, 2012) or concentrated into aggresomes if excessive. Aggresomes are depots of aggregated misfolded proteins stored in a perinuclear location as a protection mechanism from misfolded proteins (Johnston, Ward, & Kopito, 1998) and have been reported in metal‐treated cells (Sahni, Bae, Jansson, & Richardson, 2017). The aggresome–autophagy system is induced as a protein quality control mechanism. When cells are no longer stressed, the aggresome is broken down into microaggregates which can be digested by autophagy (Gao et al., 2016).

The increase in the autophagy adaptor proteins SQSTM1 and LC3‐I/LC3‐II did not correlate with a block in autophagy or with increased autophagy as there was no increase in autophagy structures (autolysosomes or early autophagic compartments). Indeed, SQSTM1, LC3‐II, and BAG3 have also been implicated in aggresome formation (Calderilla‐Barbosa et al., 2014) and were increased particularly in Cu OAC suggesting aggresome formation. In HT29, dynein, a retrograde transporter carrying aggregates to an aggresome, was increased on Cu OAC treatment. In both cell lines, further changes in heat‐shock proteins involved in the UPR (DNAJA1, HSPA1B, DNAJB1, HSPA6, and HSPH1) suggest a common mechanism of Cu OAC action.

While aggresomes are observed to have a protective role in the cell, aggresomes can interfere with proteosomal activity (Höhn & Grune, 2013) and long‐term exposure have led to DNA damage and cell cycle arrest (Lu, Boschetti, & Tunnacliffe, 2015). Evaluation of the longer‐term effect on cell growth revealed Cu OAC treatment to have a negative long‐term impact on cellular growth of Caco‐2 and may be related to aggresomes detected Cu OAC‐treated cells.

In summary, detection by DCFDA indicated differences in the ability of the organic and inorganic copper sources to stimulate ROS. Unraveling changes at the proteomic level proved most interesting with not only differential responses being noted between the two different cell lines, but also in a product‐specific fashion. Divergences were noted in terms of the cell‐specific copper uptake and in the expression of copper‐related transporters, autophagy activity, and aggresome formation. Specifically, Cu OAC appeared to induce undesirable reactions due to sustained ROS and the generation of aggresomes as a result of protein misfolding that is observed sporadically for CuSO_4_ and Cu Gly in HT29 and not observed following Cu Pro administration in either cell line. Indeed it is noteworthy, of all the copper sources tested, Cu Pro while stimulating transient ROS and higher cellular levels of copper, was the only copper source observed not to impact negatively on survival post‐treatment in both cell lines. Cu Pro is thus recommended ahead of the other copper compounds tested here (Cu Gly, CuSO_4_, and Cu OAC).

## CONFLICT OF INTEREST

The research grant supported the collaboration between Dublin City University and Alltech Ireland (IP/2015/0375). RM, KH, and IS are employed by Alltech, Ireland. All other contributors are employees of DCU. Alltech provided copper samples and ICP‐MS analysis. All other experiments were performed and interpreted by DCU authors, who have no conflict of interest.

## ETHICAL STATEMENT

Human and animal testing is not applicable to this study.
