# Supplementary material for: Acute exposure to organic and inorganic sources of copper: Differential response in intestinal cell lines
Source: Food Sci Nutr. 2018 Oct 20;6(8):2499–514. doi: 10.1002/fsn3.857 (PMC6261202; doi:10.1002/fsn3.857)
Supplement: Supplementary file 1 [file FSN3-6-2499-s001.docx]

Supplementary information

Table S1: Antibody list

| **Antibody Target** | **Abbrev.** | **Cat** | **Company** |
| --- | --- | --- | --- |
| Coppert Transporter 1 | CTR1 | ab129067 | Abcam |
| Dimetal transporter 1 | DMT1 | ab123085 |  |
| Peptide transporter 1 | PepT1 | ab123314 |  |
| Zinc Transporter | ZnT1 | ab123139 |  |
| Heme Oxygenase 1 | HMOX1 | ab13248 |  |
| metallothionein | MT1 | ab12228 |  |
| Heat shock protein 105 | HSPH1 | ab109624 |  |
| Heat shock 70 protein 6 | HSPA6 | PA5-21726 | Thermo Scientific |
| sulfidredoxin | SRXN1 | PA5-19037 |  |
| Sequestosome 1 | SQSTM1 | 5114 | Cell Signalling Technology |
| Light chain 3B | LC3B | 2775 |  |
| Ubiquitin | Ub | 3933 |  |
| Glyceraldehyde-3-phosphate dehydrogenase | GAPDH | 5174 |  |
| alpha Tubulin | aTUB | T6199 | Sigma Aldrich |
| anti-Rabbit secondary |  | A6154 |  |
| anti-Mouse secondary |  | A6782 |  |
| anti-Goat secondary A5420 |  | A5420 |  |

**Uniquely differentially expressed proteins in HT29 cells**

| **Table S2: Unique to Cu Gly in HT29** | | | | |
| --- | --- | --- | --- | --- |
| **Accession** | **Gene** | **Description** | **Fold change** | **T-test** |
| Q9Y3D9 | MRPS23 | 28S ribosomal protein S23, mitochondrial | 0 | 0.0007 |
| P22059 | OSBP | Oxysterol-binding protein 1 | 0.000 | 0.002 |
| O00159 | MYO1C | Unconventional myosin-Ic | 0.000 | 0.012 |
| P33992 | MCM5 | DNA replication licensing factor MCM5 | 0.000 | 0.048 |
| P84098 | RPL19 | 60S ribosomal protein L19 | 0.000 | 0.019 |
| O95154 | AKR7A3 | Aflatoxin B1 aldehyde reductase member 3 | 0.002 | 0.009 |
| P83731 | RPL24 | 60S ribosomal protein L24 | 0.005 | 0.007 |
| P14625 | HSP90B1 | Endoplasmin | 0.037 | 0.049 |
| O95994 | AGR2 | Anterior gradient protein 2 homolog | 0.045 | 0.001 |
| O14818 | PSMA7 | Proteasome subunit alpha type-7 | 0.047 | 0.039 |
| P62753 | RPS6 | 40S ribosomal protein S6 | 0.053 | 0.001 |
| P16104 | H2AFX | Histone H2AX | 0.068 | 0.016 |
| P49773 | HINT1 | Histidine triad nucleotide-binding protein 1 | 0.089 | 0.001 |
| Q9Y266 | NUDC | Nuclear migration protein nudC | 0.092 | 0.036 |
| P14314 | PRKCSH | Glucosidase 2 subunit beta | 0.206 | 0.019 |
| P17812 | CTPS1 | CTP synthase 1 | 0.377 | 0.042 |
| P68104 | EEF1A1 | Elongation factor 1-alpha 1 | 0.492 | 0.027 |
| P62987 | UBA52 | Ubiquitin-60S ribosomal protein L40 | 0.509 | 0.022 |

| **Table S3: Unique to Cu OAC in HT29** | | | | |
| --- | --- | --- | --- | --- |
| **Accession** | **Gene** | **Description** | **Fold change** | **T-test** |
| P62937 | PPIA | Peptidyl-prolyl cis-trans isomerase A | 0.346 | 0.035 |
| P60468 | SEC61B | Protein transport protein Sec61 subunit beta | 0.462 | 0.046 |
| O00468 | AGRN | Agrin | 0.514 | 0.000 |
| Q8WVV4 | POF1B | Protein POF1B | 0.523 | 0.005 |
| Q9UJU6 | DBNL | Drebrin-like protein | 0.549 | 0.031 |
| P09874 | PARP1 | Poly [ADP-ribose] polymerase 1 | 0.604 | 0.042 |
| P30101 | PDIA3 | Protein disulfide-isomerase A3 | 0.629 | 0.012 |
| O14737 | PDCD5 | Programmed cell death protein 5 | 0.636 | 0.039 |
| Q99623 | PHB2 | Prohibitin-2 | 0.640 | 0.035 |
| Q96EY8 | MMAB | Cob(I)yrinic acid a,c-diamide adenosyltransferase, mitochondrial | 0.641 | 0.001 |
| Q92575 | UBXN4 | UBX domain-containing protein 4 | 1.607 | 0.046 |
| Q9UHD1 | CHORDC1 | Cysteine and histidine-rich domain-containing protein 1 | 1.613 | 0.030 |
| P50454 | SERPINH1 | Serpin H1 | 1.617 | 0.025 |
| P46779 | RPL28 | 60S ribosomal protein L28 | 1.622 | 0.013 |
| Q9NQ39 | RPS10P5 | Putative 40S ribosomal protein S10-like | 1.663 | 0.021 |
| P27816 | MAP4 | Microtubule-associated protein 4 | 1.665 | 0.030 |
| P54727 | RAD23B | UV excision repair protein RAD23 homolog B | 1.751 | 0.013 |
| P28070 | PSMB4 | Proteasome subunit beta type-4 | 1.782 | 0.027 |
| P00491 | PNP | Purine nucleoside phosphorylase | 1.792 | 0.001 |
| P27635 | RPL10 | 60S ribosomal protein L10 | 1.813 | 0.041 |
| P42330 | AKR1C3 | Aldo-keto reductase family 1 member C3 | 1.827 | 0.002 |
| P62841 | RPS15 | 40S ribosomal protein S15 | 1.857 | 0.033 |
| O00148 | DDX39A | ATP-dependent RNA helicase DDX39A | 2.023 | 0.034 |
| Q71RC2 | LARP4 | La-related protein 4 | 2.023 | 0.036 |
| Q92598 | HSPH1 | Heat shock protein 105 kDa | 2.194 | 0.001 |
| P31689 | DNAJA1 | DnaJ homolog subfamily A member 1 | 2.195 | 0.002 |
| Q15181 | PPA1 | Inorganic pyrophosphatase | 2.911 | 0.006 |
| P0DMV9 | HSPA1B | Heat shock 70 kDa protein 1B | 3.212 | 0.014 |
| P25685 | DNAJB1 | DnaJ homolog subfamily B member 1 | 4.663 | 0.001 |
| Q15054 | POLD3 | DNA polymerase delta subunit 3 | 5.016 | 0.003 |
| O95817 | BAG3 | BAG family molecular chaperone regulator 3 | 7.353 | 0.008 |
| P17066 | HSPA6 | Heat shock 70 kDa protein 6 | 2.853 | 0.0004 |

| **Table S4: Unique to CuSO_4_ in HT29** | | | | |
| --- | --- | --- | --- | --- |
| **Accession** | **Gene** | **Description** | **Fold change** | **T-test** |
| O60763 | USO1 | General vesicular transport factor p115 | 1.505 | 0.033 |
| P30153 | PPP2R1A | Serine/threonine-protein phosphatase 2A 65 kDa regulatory subunit A alpha isoform | 1.510 | 0.040 |
| P05388 | RPLP | 60S ribosomal protein L8 | 1.554 | 0.030 |
| P08708 | RPS17 | 40S ribosomal protein S17 | 2.207 | 0.038 |
| Q5JRX3 | PITRM1 | Presequence protease, mitochondrial | 2.286 | 0.038 |

| **Table S5: Unique to Cu Pro in HT29** | | | | |
| --- | --- | --- | --- | --- |
| **Accession** | **Gene** | **Description** | **Fold change** | **T-test** |
| Q9Y2W1 | THRAP3 | Thyroid hormone receptor-associated protein 3 | 0.583 | 0.018 |
| Q9Y265 | RUVBL1 | RuvB-like 1 | 0.597 | 0.019 |
| Q13162 | PRDX4 | Peroxiredoxin-4 | 0.624 | 0.034 |
| P16949 | STMN1 | Stathmin | 0.633 | 0.023 |
| Q9Y2X3 | NOP58 | Nucleolar protein 58 | 1.543 | 0.043 |
| P18124 | RPL7 | 60S ribosomal protein L7 | 1.551 | 0.033 |
| P14923 | JUP | Junction plakoglobin | 1.688 | 0.040 |
| P23246 | SFPQ | Splicing factor, proline- and glutamine-rich | 1.761 | 0.044 |
| P11413 | G6PD | Glucose-6-phosphate 1-dehydrogenase | 2.013 | 0.005 |

**Uniquely differentially expressed proteins in Caco-2 cells**

| **Table S6: Unique to Cu gly in Caco-2** | | | | |
| --- | --- | --- | --- | --- |
| **Accession** | **Gene** | **Description** | **Fold change** | **T-test** |
| P31942 | USO1 | Heterogeneous nuclear ribonucleoprotein H3 | 1.7 | 0.025 |

| **Table S7: Unique to Cu OAC in Caco-2** | | | | |
| --- | --- | --- | --- | --- |
| **Accession** | **Gene** | **Description** | **Fold change** | **T-test** |
| P0DMV9 | HSPA1B | Heat shock 70 kDa protein 1B | 9.1 | 8.0E-05 |
| Q9UHD1 | CHORDC1 | Cysteine and histidine-rich domain-containing protein 1 | 1.5 | 0.012 |
| Q9NUQ6 | SPATS2L | SPATS2-like protein | 1.7 | 0.009 |
| Q9UHB6 | LIMA1 | LIM domain and actin-binding protein 1 | 1.6 | 0.028 |
| Q14247 | CTTN | Src substrate cortactin | 0.5 | 0.2 |
| P62899 | RPL31 | 60S ribosomal protein L31 | 0.6 | 0.006 |

| **Table S8: Unique to Cu Pro in Caco-2** | | | | |
| --- | --- | --- | --- | --- |
| **Accession** | **Gene** | **Description** | **Fold change** | **T-test** |
| P31689 | DNAJA1 | DnaJ homolog subfamily A member 1 | 1.7 | 0.038 |
| P13489 | RNH1 | Ribonuclease inhibitor | 0.6 | 0.006 |
| Q99959 | PKP2 | Plakophilin-2 | 0.66 | 0.022 |
| Q14192 | FHL2 | Four and a half LIM domains protein 2 | 2.42 | 0.046 |
